# Integrating 3Rs approaches in WHO guidelines for the batch release testing of biologicals: Responses from a survey of vaccines and biological therapeutics manufacturers

**DOI:** 10.1016/j.biologicals.2022.11.002

**Published:** 2023-02

**Authors:** Elliot Lilley, Emmanuelle Coppens, Pradip Das, Francis Galaway, Richard Isbrucker, Sarah Sheridan, Paul Stickings, Anthony Holmes

**Affiliations:** a NC3Rs, London, United Kingdom; b Sanofi Pasteur, France; c Biological E, India; d Medicines and Healthcare Products Regulatory Agency, United Kingdom; e WHO, Geneva, Switzerland; f Merck, United Kingdom; g National Institute for Biological Standards and Control, United Kingdom

## Abstract

The UK National Centre for the Replacement, Refinement, and Reduction of Animals in Research (NC3Rs) has been tasked by the World Health Organization (WHO) to review the extent to which animal-based testing methods are described in their manuals, guidelines and recommendations for vaccines and biotherapeutics. The aim is to identify and recommend where updates to these documents can lead to an increased and more harmonised adoption of 3Rs principles (i.e. Replacement, Reduction and Refinement of animal tests) in the quality control and batch release testing requirements for vaccines and biotherapeutics. Developing recommendations that are widely applicable by both the manufacturers and national regulatory authorities for vaccines and biologicals globally requires a detailed understanding of how different organisations view the opportunities and barriers to better integration of the 3Rs. To facilitate this, we developed and distributed a survey aimed at vaccine and biotherapeutics manufacturers in July 2021. In this paper, we present the key findings from this survey and how these will help inform the recommendations for wider integration of 3Rs approaches by WHO in their guidance documents applicable to the quality control and batch testing of vaccines and biotherapeutics.

## 1 Introduction

Vaccines and biotherapeutics are products derived from biological sources. Due to the inherent heterogeneity present in biological systems, individual batches of vaccines and biotherapeutics are tested at multiple stages during manufacture and at the end of each production run in order to ensure consistency with prior batches and to provide assurance for safe and effective clinical use. Vaccines and biological therapeutics are important for human health and it is essential that critical quality attributes are tightly controlled. The World Health Organization (WHO) is mandated to establish international norms and standards for this purpose and as such their guidelines and recommendations carry significant influence, being adopted by most global regulatory authorities. The UK National Centre for the Replacement, Refinement, and Reduction of Animals in Research (NC3Rs) has been tasked by the WHO to review animal-based testing methods used for quality control purposes as described in WHO written standards for vaccines and biological therapeutics to identify opportunities for increased and more harmonised adoption of 3Rs principles (i.e. Replacement, Reduction, and Refinement of animal tests) [1].

A critical aspect in delivering this ambition is to engage with relevant communities to better understand the opportunities and challenges faced in greater implementation of 3Rs approaches in this testing. The two key stakeholder groups in this effort are the biologicals manufacturers and the regulatory authorities responsible for developing and ensuring safe and efficacious products are available for consumer use. To understand their perspective on this issue the NC3Rs has surveyed both communities. In this paper, the key findings from the manufacturer survey are presented. Regulatory perspectives will be described in a separate publication.

## 2 Survey information (method)

The survey was distributed as a Microsoft Excel™ file and consisted of three sections:

1. Demographic data,
2. Questions regarding current practices with respect to animal-based methods for quality control and batch release testing of vaccines and biologicals,
3. Questions regarding opportunities and barriers to the adoption of 3Rs and non-animal methods used in the quality control and batch release testing of vaccines and biologicals.

Prior to distribution, the survey passed ethical review by the Royal Veterinary College (RVC, London, UK) Social Science Research Ethical Review Board (RVC ref: URN SR2021-0131). It was launched in July 2021 and formally closed in September 2021 (though a small number of responses were received after this date and are included in this paper). The survey was distributed (via a link to the NC3Rs website) through advertising on social media, direct email to networks and industry newsletters and websites. We received 30 complete responses from 25 different vaccine manufacturers and allowed multiple submissions from multi-national manufacturers where these were from distinct subsidiaries or country locations. The nature of the survey dissemination means that we do not know how many individual manufacturers were aware of the survey and therefore, we cannot judge the overall response rate. The questions from the survey are reproduced in Appendix 1.

## 3 Survey data (results)

The survey data received was fully anonymised prior to its analysis. All product and animal test data were randomised and any information that could identify individuals or their employers was redacted. The anonymised dataset was shared with an international group of experts in the production, regulation and quality control of biologicals to support data analysis and interpretation and who are co-authors of this paper. The anonymised data from the survey analysis is available as supplementary materials (file: Supplementary materials_manufacturers survey raw data.xslx).

### 3.1 Survey respondent demographics

30 complete responses were received from 25 manufacturers in 16 different countries. The majority (21/30) were from respondents based in the Asia-Pacific region; with the remainder of the responses from manufacturers located in Europe (7/30), North America (1/30) or South America (1/30). Half of the respondents (15/30) held managerial positions in their organisations although a wide range of occupations were represented including bioanalyst, study director and veterinarian.

The regional and country information collected in this survey should be viewed with a significant caveat. Many vaccine and biologicals manufacturers operate across several countries and the survey respondents gave the location that they were responding from. It was unclear from the survey responses whether questions were answered from a global, regional, or individual perspective. The working group decided that, on this basis, they could not make any region or country related conclusions or correlations with subsequent data and that the demographic data should be provided for information only.

### 3.2 Product information

A significant amount of product-related data was provided by the survey respondents. Respondents were asked: ‘What vaccines product, vaccine component or biological products do you produce?’ 154 individual product types were reported (Table 1). For each product, respondents were asked to provide details about the quality control/batch release tests performed using animals. 416 individual animal tests spanning 20 different animal-based method subcategories were reported (Table 2). Respondents were also asked to indicate whether they were aware of, were exploring, or had adopted suitable non-animal technologies (NATs) for any of these assays (Table 2).

**Table 1:** List of products produced by manufacturers responding to the survey.

Products by type	Number of respondents reporting they manufacture the product
Adjuvants	3
Antiserums and antivenoms	8
Bacillus Calmette-Guérin (BCG) vaccine and BCG-related products	9
Biotherapeutics and monoclonal antibodies	7
Cell banks, seed stocks	3
COVID-19 vaccines	4
Diphtheria, Tetanus & Pertussis (DTP) containing vaccines	32
Ebola vaccine	1
Hepatitis A vaccines	6
Hepatitis B vaccines	9
Hepatitis E vaccines	2
Human papillomavirus (HPV) vaccines	3
Influenza vaccines	7
Japanese encephalitis vaccines	5
Misc vaccines	6
Measles, mumps, and rubella (MMR) vaccines	15
Multi-product entries	6
Polio vaccines	10
Polysaccharide and conjugate vaccines	7
Rabies vaccines	9
Tick-borne encephalitis vaccines	2
**Total products**	**154**

**Table 2:** List of animal-based methods performed by manufacturers responding to the survey.

Method subcategory	Number of times animal tests reported	% of respondents aware of/or exploring NATs	% of respondents that have already replaced animal tests with NATs
Potency	121	57.9	15.7
ATT^a^	86	1.2	17.4
Adventitious agents	47	38.3	2.1
Specific toxicity	45	21.5	11.1
Pyrogen test	39	92.3	46.2
Detoxification	18	11.1	5.6
Irreversibility/reversion test	18	44.4	5.6
Mouse Weight Gain	11	0.0	0.0
Neurovirulence test	6	33.3	0.0
Leukopenic toxicity	5	0.0	0.0
Other^b^	20	10.0	0.0
Total	416		

a Note that the ATT/GST/innocuity test does not have an alternative non-animal replacement. It is widely acknowledged that this test is not scientifically justifiable and no longer relevant or needed and has been removed from many pharmacopeia and is no longer recommended in WHO guidelines rather than replaced. Respondents who have indicated that they know of, or use, an NAT for this test are most likely misinterpreting the question.

b Other tests (with less than 5 reports) consists of the following tests: Dermal reactivity, Safety test, Marker test, Attenuation test, Whole cell pertussis – dermonecrotic toxin (wP – DNT), Inactivation, Identity, Leachable test, Stability.

Respondents were asked to provide information about the species and number of animals used. However, it was not possible to determine the number of individual animals used in total per year as respondents were not asked to report how many batches they produced annually for each product. The species most commonly used for these tests were mice (45%), guinea pigs (40%) and rabbits (10%).

Awareness of NATs was variable overall but there were clear subcategories of tests where knowledge of alternative approaches was more prevalent than others. For example, nearly all respondents (92%) were aware of alternative approaches for pyrogen testing, with the monocyte activation test (MAT) most mentioned. Awareness of alternatives for potency, toxoid reversion, irreversibility and adventitious agents testing was reasonably high (58%, 50%, 42% and 38% respectively). It is notable that for some products, awareness of non-animal potency tests was very high. For example, awareness of alternative potency testing approaches for hepatitis B and E vaccines, rabies vaccines and polio vaccines was very high (100%, 89% and 80% respectively). However, in general, current implementation of NATs was very low (apart from pyrogen testing with 46% implementation). This may be due to the way the question was framed – ‘What quality control/batch release tests do you perform in animals for each product listed?” – this specifically referred to tests that were currently performed in animals and may have been taken by some respondents as excluding NATs. The data shows a clear disconnect between *awareness* and *implementation* of NATs. A range of factors likely contribute to this which will be explored in the 3Rs section below.

### 3.3 Current practice in NATs

In addition to asking about specific product-related animal tests, a series of questions were asked about current practices regarding adoption and use of NATs.

**Fig. 1:**
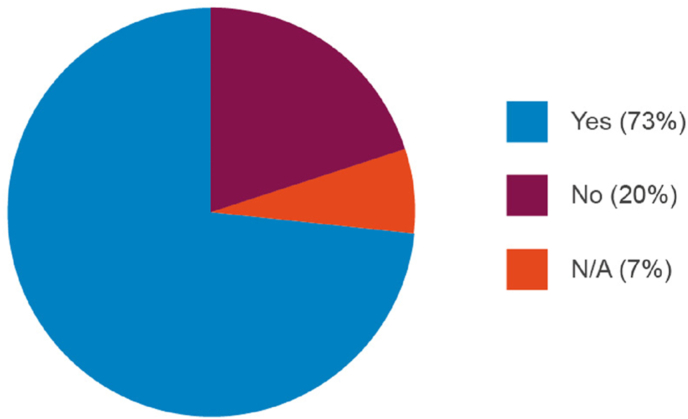
Proportion of manufacturers that have discussed adoption of non-animal alternative approaches for quality control and batch release testing with a regulator.

**Fig. 2:**
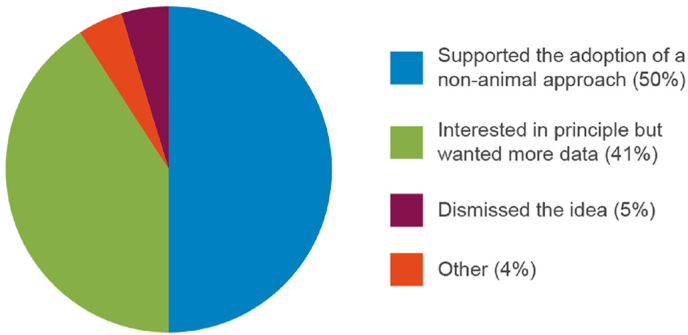
The outcome of discussions with regulators regarding adoption of non-animal technologies for quality control and batch release testing.

Encouragingly, most respondents (73%; Fig. 1) had discussed the potential use of NATs with National Regulatory Authorities (NRA) who were broadly supportive of this change from *in vivo* to *in vitro* testing (37% of responses stated that the NRA supported the use of NATs, 30% stated that the NRA was interested in principle but wanted more data; Fig. 2).

Less encouraging was the extent to which NATs are currently used by the manufacturers who responded to the survey. The survey asked if their testing approach varied depending on the region in which products were released following marketing authorization. Specifically, to establish if they used NATs for regions where this would be accepted by regulators or if they only use animal tests because they are more widely accepted. 32% of respondents indicated ‘Yes, we conduct non-animal tests for marketing in those regions where regulators will accept the data and animal tests for all other regions’ whereas the majority (66%) of respondents answered ‘No, we conduct only the animal studies as these are acceptable for all regional regulators’.

**Fig. 3:**
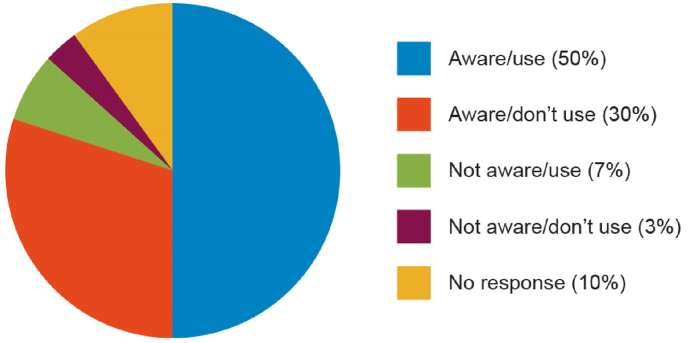
Awareness of the WHO decision to remove the ATT from their requirements and whether manufacturers who responded to the survey still perform the test.

The Abnormal Toxicity Test (ATT) (also referred to as the General Safety Test (GST) or test for innocuity) has been widely acknowledged as being scientifically questionable, non-reproducible and non-specific [2,3]. The WHO Expert Committee on Biological Standardization (ECBS) followed the United States of America Food and Drug Administration (FDA), the European Pharmacopeia (Ph Eur) and others in removing this test from their requirements in 2018 [4]. However, despite this, it is clear that the ATT is still widely requested by regulatory authorities and/or used by manufacturers globally. To understand better the reasons behind this, respondents were asked if they were aware that the WHO had removed the requirement to perform this test and whether they still performed the test as part of their quality control and batch release testing. There was good awareness that the WHO had removed this test (80%) but despite this, most manufacturers who completed the survey (57%) still perform the test (Fig. 3).

This may be because many of the manufacturers who responded to the survey market their products to multiple regions, some of which still require the ATT to satisfy their national regulations/requirements. This lack of global harmonisation with respect to requirements to perform this test is a critical factor in its continued use. Additionally, the slow removal of the ATT may be due to the need to submit variations for licensed products to have this test removed - regulators may not require the test, but suppression of the test is not a passive process.

### 3.4 3Rs awareness and practice

Based on the extensive interaction the NC3Rs has had with the scientific community over many years, it is likely that a significant barrier to the adoption of 3Rs approaches is a lack of awareness that appropriate non-animal technologies are available. We wanted to explore if this was also the case for the vaccines and biological therapeutics community to better understand how best to support the adoption of these approaches.

**Fig. 4:**
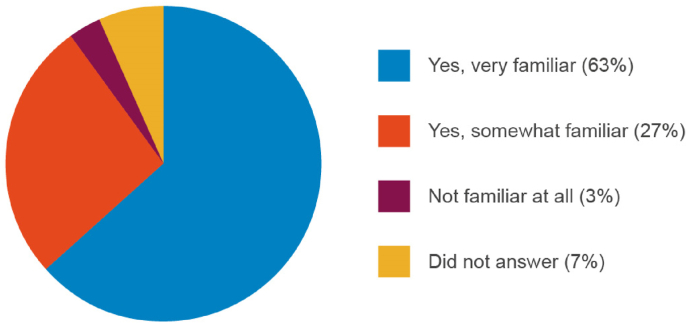
Awareness of the 3Rs by manufacturers.

**Fig. 5:**
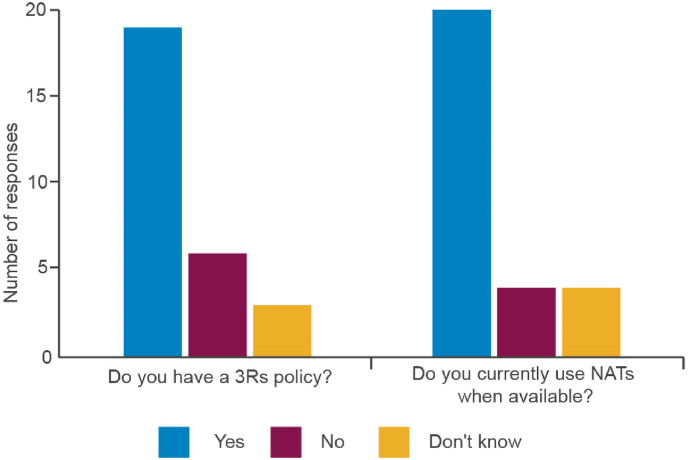
Further information regarding the implementation of the 3Rs.

Almost all manufacturers (90%) were very or somewhat familiar with the 3Rs (Fig. 4) and 60% stated that their organization had a 3Rs policy. Interestingly, 64% indicated that they used NATs when they were available (Fig. 5). This could be viewed as in conflict with the response to question 2c where only 32% of respondents indicated that they use NATs for markets where this would be acceptable. However, it is likely that the issue of global acceptance of NATs is considered when judging whether a NAT is ‘available’ or viable as an alternative testing method. Also, for a product marketed across several regulatory jurisdictions, the continued use of an *in vivo* method may be required, despite an alternative being available, if even a single regulatory authority still requires it.

**Fig. 6:**
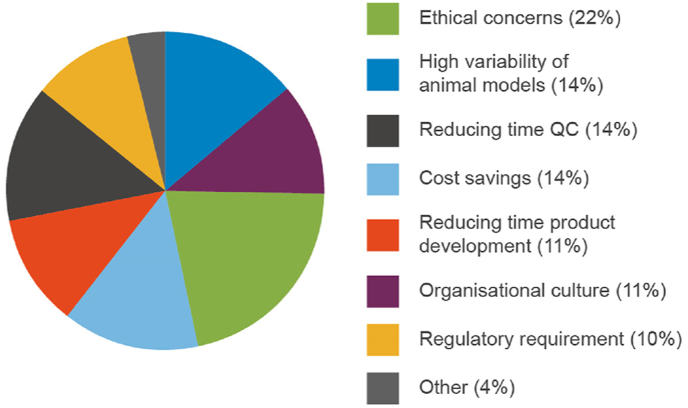
Factors that drive adoption of NATs in quality control and batch release testing.

Respondents that indicated they used NATs when they were available (64%), were asked what drivers influenced this decision (question 3d in Appendix 1). The survey allowed respondents to select multiple drivers with the most frequently (expressed as a percentage of the total responses; 79 total responses were given) chosen being ethical concerns for animal welfare (22%), reducing time for quality control testing (15%) and the high variability of data generated in animal models (13%; Fig. 6).

**Fig. 7:**
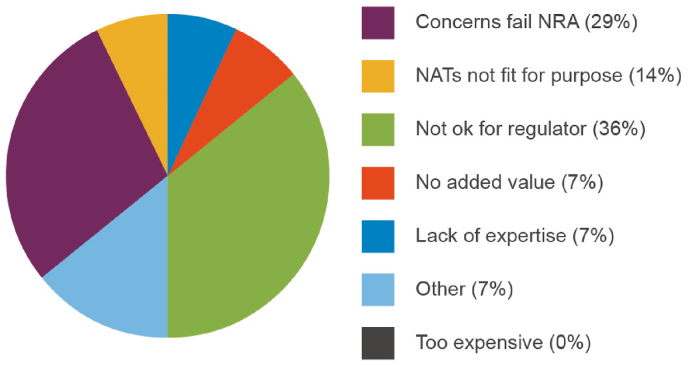
Factors that prevent adoption of NATs in quality control and batch release testing.

Respondents that indicated that they did not use NATs were asked what the barriers were which prevented them from doing so (question 3e in Appendix 1). Again, the survey allowed respondents to select multiple barriers with the most frequently (expressed as a percentage of the total responses; 14 total responses were given) chosen being concerns that data generated would not be accepted by regulators (34%) and concerns that regulatory requirements would not be met (34%; Fig. 7).

**Fig. 8:**
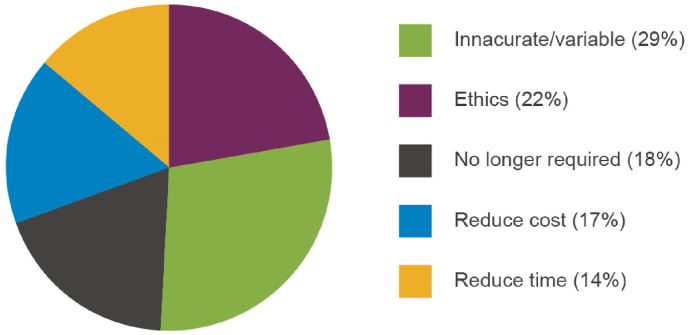
Reasons given to remove or replace existing *in vivo* tests.

Understanding the validity of existing tests is important in determining the likelihood of uptake of alternative approaches. Respondents were asked if there were any currently used animal methods that were not ‘fit for purpose’ (68% indicated that this was the case) and whether these should be replaced/removed from use (Question 3 h in Appendix 1). Several current *in vivo* tests were highlighted as not being fit for purpose and a diverse range of reasons were given by respondents to support this. These fell into five broad categories (Fig. 8) with issues around variability/accuracy of animal models and ethics/animal welfare concerns ranking most highly.

Of the 67 animal tests that were reported as being desirable for potential replacement or removal, there was a good awareness of potential NATs that could be used as replacements (64%) and half (49%) of respondents indicated that they had plans to adopt NAT approaches (Table 3).

**Table 3:** Availability of NATs for tests identified for removal or replacement and whether manufacturers plan to remove them.

NAT available?		Plan to remove or replace		% know of NAT	% plan to remove or replace
All Tests					
Yes	45	Yes	35	**65**	**51**
No	21	No	31		
Don't know	3	Don't know	3		
Total	69		69		
**Potency**					
Yes	26	Yes	17	**70**	**46**
No	8	No	19		
Don't know	3	Don't know	1		
Total	37	Total	37		
**Abnormal toxicity**					
Yes	0	Yes	1	**0**	**17**
No	6	No	5		
Don't know	0	Don't know	0		
Total	6	Total	6		
**Pyrogen testing**					
Yes	5	Yes	5	**100**	**100**
No	0	No	0		
Don't know	0	Don't know	0		
Total	5	Total	5		
**Safety testing**					
Yes	8	Yes	8	**57**	**57**
No	6	No	4		
Don't know	0	Don't know	2		
Total	14	Total	14		
**Adventitious agents testing**					
Yes	4	Yes	2	**100**	**50**
No	0	No	2		
Don't know	0	Don't know	0		
Total	4	Total	4		
**Neurovirulence testing**					
Yes	0	Yes	0	**0**	**0**
No	1	No	1		
Don't know	0	Don't know	0		
Total	1	Total	1		

There is significant global activity and investment in NATs but ensuring these are appropriate for quality control and batch release testing purposes can be difficult. One approach to address this is to establish in-house programmes for model development. It is encouraging that 50% of respondents indicated that they currently had a dedicated in-house program to develop and/or validate NATs.

**Fig. 9:**
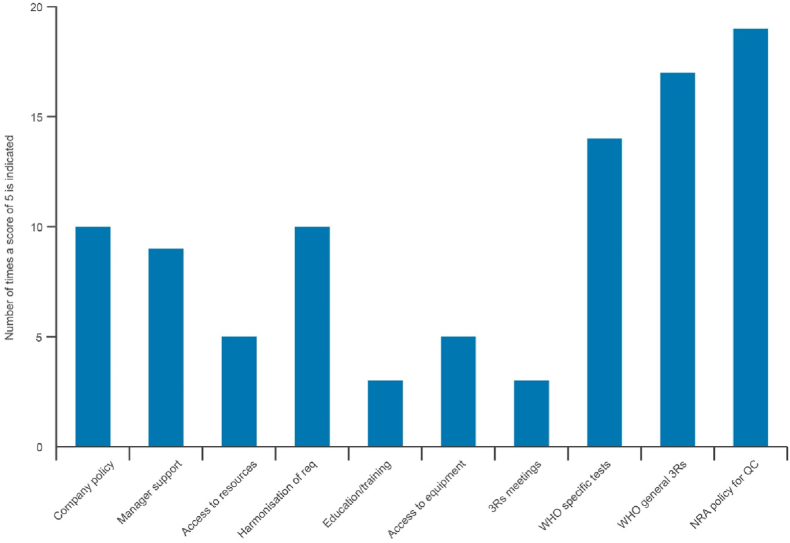
The highest rated factors that would support increased adoption of the 3Rs in quality control and batch release testing.

Understanding what factors would support manufacturers to further adopt 3Rs approaches in their quality control and batch release testing programmes is an important aspect of this project. Respondents were given the opportunity to select from 11 options and rank these (from 1 - least important to 5 - most important) for the impact they would have on the adoption of 3Rs approaches within their organization (Question 3j in Appendix 1). The options selected most frequently as important were changes to WHO guidance for specific products, general guidance from the WHO on adoption of the 3Rs, and changes to NRA policy to promote 3Rs for quality control of biologics (Fig. 9).

## 4 Discussion

Historically, many tests used in a quality control strategy for the assessment of batch to batch safety, efficacy and production consistency for vaccines and biological therapeutics have involved animals, although in recent years NATs, better quality control measures (such as in-process controls, an understanding of critical quality attributes, and implementation of consistency approaches in manufacturing) are increasingly resulting in control strategies that have reduced or eliminated routine animal test methods [[5], [6], [7], [8], [9]]. It is also important to highlight that for quality control and batch release purposes, NATs are considered superior to animal tests due to improved specificity and improved precision which makes them less variable and better suited for the purposes of batch-to-batch QC testing. These non-animal methods also bring additional advantages in terms of reduced duration for testing (which is an important factor in ensuring supply of medicines) and reduced cost [14,15]. For example an animal vaccine potency test may require weeks to months after administration to determine the potency and involve animal care and laboratory staff whereas a suitable NAT to confirm the vaccine antigen content and quality may take just days to conduct by a single individual. In addition to the robust scientific argument to transition away from animal testing paradigms there are also ethical and animal welfare concerns as the methods themselves can cause significant pain and distress to the animals [[10], [11], [12], [13]].

This survey set out to gain greater understanding around how animals are used by vaccine and biologicals manufacturers in quality control and batch release testing and to explore barriers and opportunities for greater implementation of the 3Rs [1]. Animals are still widely used – 416 animal tests spanning 20 different animal-based methods for 154 products were reported by the 30 manufacturers who responded to the survey. It should be noted that, despite a concerted effort to promote the survey globally, the overall low response rate and geographical distribution of responses brings significant caveats to the global applicability of the data and conclusions drawn from these data. Although there was good awareness of NATs in many test categories, NAT use was very low overall. This may be due to the way the question (2a in Appendix 1) was worded; we asked: “what quality control/batch release tests do you perform in animals for each product listed”. Products where NATs are already being used may not have been reported. The most common test categories where animals are used consistently were potency (29.3%) followed by ATT/GST (20.5%), adventitious agents (11.2%), specific toxicity (11%) and pyrogen testing (9.3%). It is particularly interesting that the ATT/GST test is still widely used given that the WHO [4] and several National pharmacopeia [3, [16], [17], [18]] have deleted the requirement for this test from their guidelines. We specifically asked whether respondents were aware that the WHO had deleted the ATT/GST and nearly 80% indicated that they were aware of the decision of ECBS in 2018. We postulate that there are three potential reasons for the continued use of the ATT/GST despite this. Firstly, that some countries have yet to delete this test from their national pharmacopeia and the lack of global harmonisation with respect to regulatory requirement for the ATT/GST means that vaccine manufacturers which market to multiple countries will still perform the test. Secondly, although the WHO no longer requires the ATT/GST (as approved by ECBS [4]), many individuals WHO guidelines have yet to be updated to remove the test from the written, published text. Thirdly, that even when a regulatory authority no longer requires the ATT, the individual product licence has to be updated via a post approval change [19] to remove the test. This is not a straightforward process and can cause significant delays in the release of products. While current adoption of NATs may be low, there is a clear appetite from manufacturers to engage with regulatory authorities on how to transition away from animal tests because they recognise the scientific, commercial and animal welfare benefits this offers. Most manufacturers who completed the survey indicated that they have engaged with regulators on this and that these discussions were positive, although in some cases more data was needed to convince regulators of the validity of the NATs presented.

Overall awareness of the 3Rs was very high reflecting the fact that, following many years of promotion by organisations like the NC3Rs, these principles are now part of the common lexicon of science communication and policy. Although the ethical concerns over animal use are a factor for implementation of the 3Rs, there are clear, robust scientific arguments for transitioning away from animal testing approaches to NATs. Respondents indicate that they believed that animal tests were more variable, more expensive and more time consuming to run than NATs. We believe that, whilst the ethical argument is important, these scientific and commercial benefits are more likely to stimulate global adoption of NATs.

This survey was one of several stakeholder engagement activities (including a separate survey to biologicals regulators and regional stakeholder workshops [20]; conducted as part of a project to review animal testing requirements within WHO guidelines [1]. All relevant, publicly accessible WHO guidance documents [21] for the quality, safety and efficacy of vaccines and biologicals will be reviewed, and recommendations made to update these guidelines to promote more harmonised adoption of 3Rs principles in biologicals batch release testing. It is reassuring then that most respondents indicated that such revisions would help them to apply 3Rs approaches in the future.

In conclusion, the manufacturers survey data indicates that, whilst awareness of the 3Rs and the potential to use NATs to replace animal use in quality control and batch release testing of biologicals is high, the current rate of change in this direction is slow and more needs to be done to accelerate progress. The challenges in achieving this goal should not be underestimated but it is clear from this survey that there is an appetite for change. Indeed, it is highly encouraging that half of the manufacturers who responded to this survey indicated that they had dedicated in-house programs to develop and/or validate NATs. Transitioning away from animal test methodologies can benefit from harmonisation and cooperation between manufacturers and between manufacturers and regulators. NATs need to be standardised wherever possible with protocols and reagents that are easy to follow and affordable. This survey suggests that most vaccine and biological therapeutics manufacturers see the benefits of NATs for quality control and batch release testing of biologicals and are willing to replace current animal-based testing methodologies, this may take time and effort to fully realise but positive engagement and a willingness to embrace NATs is an important first step.
